# Correlates of employment status in individuals with asthma: a cross-sectional survey

**DOI:** 10.1186/s12995-017-0165-6

**Published:** 2017-07-24

**Authors:** Saara Taponen, Lauri Lehtimäki, Kirsi Karvala, Ritva Luukkonen, Jukka Uitti

**Affiliations:** 1 Finla Occupational Health, Satakunnankatu 18 B, 33210 Tampere, Finland; 2 Allergy Centre, Tampere University Hospital, PO Box 2000, 33521 Tampere, Finland; 3 Faculty of Medicine and Life Sciences, University of Tampere, 33014 Tampere, Finland; 4 Finnish Institute of Occupational Health, PO Box 40, 00251 Helsinki, Finland; 5 Clinicum, Faculty of Medicine, University of Helsinki, PO Box 63, 00014 Helsinki, Finland

**Keywords:** Asthma symptoms, Asthma, Work ability, Work

## Abstract

**Background:**

This study aims to elucidate factors that among adults with asthma are associated with working full-time.

**Methods:**

This cross-sectional survey of 2613 working-age adults with asthma included questions on asthma history, symptoms and use of asthma medication, socioeconomic factors and health behavior. Full-time workers were compared to groups according to employment status: unemployed, work disability and retired due to age.

**Results:**

Adults with asthma working full time were younger and more often nonmanual workers, experienced less asthma symptoms, used less asthma medication and smoked less than subjects with work disability. After adjusting for age, gender, smoking and professional status, having frequent symptoms of asthma during last month was associated with an increase in the risk of unemployment (OR 2.3, 95% CI 1.3–4.2) and with an increase in the risk of work disability (OR 4.4, 95% CI 2.3–8.2).

**Conclusions:**

Among adults with asthma, full-time work was associated with younger age, less symptomatic asthma despite of less medication, nonmanual work and less smoking. Having more severe symptoms of asthma was associated with undesirable employment status such as unemployment or work disability. Possibilities to change from manual to nonmanual work may be important in preventing work disability and early exit from work.

**Electronic supplementary material:**

The online version of this article (doi:10.1186/s12995-017-0165-6) contains supplementary material, which is available to authorized users.

## Background

Asthma is a common chronic disease among working age adults with prevalence in Finland as high as nearly 10% [1] and globally, around 4.5% [2]. Work disability of adults with respiratory conditions such as asthma is continuously a major concern due to its significant economic burden [3]. Unemployment rate among individuals with asthma is high, sick leave from work is more common among individuals with asthma than without asthma [4] and return to work takes more time from individuals with asthma especially among blue-collar workers. In cases of occupational asthma, job loss seems to be even more likely with less education [5]. In one prospective study, adults with severe asthma were 31% less likely to be currently employed [6].

Respiratory work disability has been defined in many ways, e.g. as job changes due to respiratory problems, sickness absences, or in its most serious form, complete cessation of employment due to respiratory problems [7]. In general, work disability is associated with working conditions, socioeconomic factors such as lower level education, and health behavior such as smoking [8]. Based on earlier studies, workplace exposure to dusts or other impurities is associated with work-related respiratory symptoms and disability [9] and among asthmatics, increased respiratory sickness absence [10], work disability [6] and exacerbation of asthma symptoms [11]. In individuals with asthma, higher educational attainment indeed seems protective whereas a history of smoking associates with a greater risk of work disability [6, 12].

Severity of asthma strongly predicts work disability among adults with asthma [6, 12, 13]. Severity of asthma also decreases the quality of life of asthma patients, as also do lower education, presence of stressful events and a poor control of asthma [14]. A survey study in the U.S. compared the employment status of subjects with work-related and non-work-related asthma and factors associated with unemployment. The results showed that those who were unable to work were more likely to have more frequent asthma symptoms, very poorly controlled asthma and increased health care utilization than their counterparts [15]. Comorbidity plays a major role as was shown in a prospective population-based study by Hakola et al. where the risk of all-cause long-term work disability increased in the presence of asthma [16].

Based on these studies it is apparent that subjects with asthma may have more difficulties in working life than subjects with no asthma. However, some adults with asthma cope well in working life while others face problems. Identifying the factors that support full-time work and holding on to a job among adults with asthma is crucial in order to support their working careers. These factors likely predict how adults with asthma cope in working life, i.e. who succeed to continue as full-time workers and who drift away from active working life due to e.g. sickness absence, disability pension, early retirement or unemployment.

The aim of this study was to investigate which characteristics of asthma are associated with employment status. Using full-time work as an indicator of coping well in working life, we assessed how asthma symptoms, use of asthma medication and socioeconomic factors are related to employment status among adults with asthma in a general population.

## Methods

This cross-sectional survey study recruited all asthma patients (*n* = 2613) aged 20–65 years and living in the city of Tampere (total population 190,000), Finland. The cases were identified from the Medication Reimbursement Register of the Finnish Social Insurance Institution. All those who had been granted special reimbursement rights for asthma medication until the end of 1997 and were alive in October 2000 were selected. To be granted reimbursement rights by the Finnish Social Insurance Institution, the disease must fulfill the diagnostic and severity criteria of asthma, including objective data of reversible/variable bronchial obstruction and a need for regular treatment with inhaled glucocorticoids for at least 6 months after the diagnosis. Among those granted special reimbursement rights for asthma medication, the reliability of the asthma diagnosis is high [11]. The questionnaire was sent in October 2000 and the response rate was 79%. Ninety eight subjects were excluded from the analyses because it was double-checked that the respondents indeed have asthma diagnosed by a physician (some may have had asthma-COPD overlap) or because the employment status information was missing. For this study, the respondents were divided into five groups according to their working life status. There were 967 subjects working full-time, 197 unemployed subjects, 334 subjects with work disability (including all-cause sickness absence, disability pension, and disability pension applied but not yet granted) and 159 subjects retired due to age (in Finland, age-related pension starts at age of approximately 60–68 years depending on occupation and personal preferences). The fifth group consisting of subjects outside of full-time work for other reasons (e.g. housewives, students, part-time workers, maternity leave, etc.) were excluded from this study (*n* = 309).

### Questionnaire

The self-administered questionnaire included questions on asthma history, asthma symptoms and use of asthma medication, professional status, smoking, and leisure time activities. Additional file 1: Appendix 1 lists the specific questions of the postal questionnaire that were used in this study.

### Statistical analyses

In our study, we were especially interested in whether the full-time workers differ from the subjects in other three groups (unemployed, those with work disability and those retired). Our data set consisted of both continuous and categorical variables. When comparing the differences between groups we applied ANOVA with Dunnet’s post-test (variances were not equal between the groups) for a continued variable age and Chi-squared tests for categorical variables.

After these preliminary studies we built logistic regression models using either unemployed vs. full-time work or work disability vs. full-time work as an outcome variable. Different asthma symptoms and information of asthma medication were used as independent variables one at a time. Our model building strategy was as follows. At first we estimated crude models and then adjusted models with age, gender, smoking, and professional status. The odds ratios with 95% confidence intervals only for the highest levels (“worst” vs. “none”) have been presented in Table 3. A *p*-value of <.05 was considered statistically significant. All analyses were carried out using SPSS (version 23) program.

## Results

Table 1 shows the characteristics of subjects in four different groups divided according to their status in working life.

**Table 1:** Characteristics according to working life status

	Full-time work (1) *n* = 967	Unemployed (2) *n* = 197	Work disability (3) *n* = 334	Retired (4) *n* = 159	*p*-value 1 vs. 2	*p*-value 1 vs. 3	*p*-value 1 vs. 4
Age, mean years (SD)	44.1 (10.2)	46.2 (11.0)	57.9 (8.2)	62.9 (3.8)	0.064	<.001	<.001
Gender					0.007	0.346	0.028
Women	568 (58.7)	136 (69.0)	206 (61.7)	108 (67.9)			
Men	399 (41.3)	61 (31.0)	128 (38.3)	51 (32.1)			
Smoking status					0.004	0.002	<.001
Never smoker	432 (45.0)	70 (36.1)	132 (40.0)	92 (58.2)			
Ex-smoker	217 (22.6)	40 (20.6)	98 (29.7)	38 (24.1)			
Current smoker	155 (16.1)	52 (26.8)	66 (20.0)	20 (12.7)			
Occasional smoker	157 (16.3)	32 (16.5)	34 (10.3)	8 (5.1)			
Professional status					<.001	<.001	0.001
Self-employed	98 (10.2)	10 (5.2)	25 (8.0)	7 (4.6)			
Upper level nonmanual worker	199 (20.7)	11 (5.8)	21 (6.7)	24 (15.9)			
Lower level nonmanual worker	284 (29.5)	50 (26.2)	59 (18.8)	35 (23.2)			
Manual worker	353 (36.7)	113 (59.2)	195 (62.1)	79 (52.3)			
Working from home, student, other	28 (2.9)	7 (3.7)	14 (4.5)	6 (4.0)			
Physical exercise frequency					0.133	<.001	0.007
Not at all	50 (5.2)	14 (7.3)	33 (10.1)	6 (3.8)			
Less than once a week	224 (23.3)	38 (19.7)	60 (18.3)	24 (15.3)			
1–2 times a week	423 (44.0)	75 (38.9)	125 (38.2)	64 (40.8)			
> = 3 times a week	265 (27.5)	66 (34.2)	109 (33.3)	63 (40.1)			

Data is presented as n (%) unless otherwise stated

The groups consisting of subjects working full time and of those unemployed were youngest and those with work disability and those retired were oldest. There were more women than men in all groups but the proportion of women was greatest among the unemployed and the retired.

### Smoking and physical exercise

The smoking habits of full-time workers were overall lighter than in the work disability group. Among the unemployed, the proportion of current smokers was greatest and of never-smokers smallest of all groups. The proportion of ex-smokers was greatest in the work disability group. Smoking in general was less common in the retired group: there were less current smokers and occasional smokers and more never-smokers in the retired group than in the full-time work group. The groups were quite similar according to physical exercise frequency. Full-time workers reported less often no exercise at all than subjects with work disability.

### Professional status

There were more upper level (20.7 vs. 6.7%) and lower level (29.5 vs. 18.8%) nonmanual workers and less manual workers (36.7 vs. 62.1%) in the full-time working group compared to the work disability group, respectively. Compared to the unemployed group, there were more upper level nonmanual workers and less manual workers in the full-time working group. Also in the retired group, there were more manual workers (52.3 vs. 36.7%) and less nonmanual workers 39.1 vs. 50.2%) than in the full-time working group.

### Asthma symptoms and medication

Table 2 shows the occurrence and frequency of asthma symptoms and the use of asthma medication in different employment status groups.

**Table 2:** Asthma symptoms in groups according to employment status

		Full-time work *n* = 870–960	Unemployed *n* = 193	Work disability *n* = 309–327	Retired *n* = 153	*p*-value 1 vs. 2	*p*-value 1 vs. 3	*p*-value 1 vs. 4
Occurrence of asthma symptoms during last year	Persistently	257 (26.8)	73 (37.8)	189 (57.8)	69 (45.1)	0.003	<.001	<.001
	Seasonally	162 (16.9)	34 (17.6)	55 (16.8)	26 (17.0)			
	Occasionally	450 (46.9)	78 (40.4)	66 (20.2)	42 (27.5)			
	None	91 (9.5)	8 (4.1)	17 (5.2)	16 (10.5)			
Frequency of asthma symptoms during last month	Daily or almost daily	93 (10.7)	39 (21.3)	139 (45.0)	38 (27.7)	<.001	<.001	<.001
	3–5 times a week	71 (8.2)	14 (7.7)	38 (12.3)	12 (8.8)			
	1–2 times a week	176 (20.2)	45 (24.6)	44 (14.2)	26 (19.0)			
	Less than once a week	336 (38.6)	58 (31.7)	58 (18.8)	40 (29.2)			
	None	194 (22.3)	27 (14.8)	30 (9.7)	21 (15.3)			
Frequency of asthma symptoms during last year	Daily or almost daily	121 (14.6)	46 (27.2)	113 (42.3)	37 (30.3)	<.001	<.001	<.001
	3–5 times a week	84 (10.1)	23 (13.6)	44 (16.5)	16 (13.1)			
	1–2 times a week	187 (22.6)	41 (24.3)	46 (17.2)	23 (18.9)			
	Less than once a week	415 (50.1)	57 (33.7)	58 (21.7)	40 (32.8)			
	None	22 (2.7)	2 (1.2)	6 (2.2)	6 (4.9)			
Nightly wake-ups because of asthma symptoms during last month	> = 3 times a month	162 (18.4)	72 (39.1)	170 (55.4)	49 (34.8)	<.001	<.001	<.001
	<3 times a month	191 (21.7)	34 (18.5)	50 (16.3)	31 (22.0)			
	None	526 (59.8)	78 (42.4)	87 (28.3)	61 (43.3)			
Nightly wake-ups because of asthma symptoms during last year	> = 3 times a month	151 (17.8)	61 (35.3)	129 (46.2)	34 (27.4)	<.001	<.001	0.004
	<3 times a month	396 (46.7)	69 (39.9)	99 (35.5)	62 (50.0)			
	None	301 (35.5)	43 (24.9)	51 (18.3)	28 (22.6)			
Use of asthma medication during last year	No	105 (10.9)	13 (6.6)	19 (5.7)	12 (7.6)	0.066	<.001	0.010
	Irregularly	190 (19.7)	39 (19.9)	38 (11.5)	18 (11.4)			
	Seasonally (e.g. springtime)	113 (11.7)	15 (7.7)	23 (6.9)	15 (9.5)			
	Continuosly daily or almost daily	556 (57.7)	129 (65.8)	251 (75.8)	113 (71.5)			
Subjects having used peroral corticoids because of asthma during last year		225 (23.5)	53 (27.3)	143 (43.7)	41 (26.6)	0.258	<.001	0.401

Data is presented as n (%)

Full-time workers experienced altogether least asthma symptoms: year-round symptoms, daily symptoms and wake-ups at night were less common among full time workers than among other groups, of whom persons with work disability seemed to be the most symptomatic. For example, 10.7% of full time workers vs. 45.0% of those with work disabilities experienced asthma symptoms daily or almost daily during the last month. While having less symptoms of asthma, full-time workers used less medication for asthma than subjects with work disability. Use of peroral corticoids was more common among persons with work disability than among full-time workers.

Table 3 shows the associations between asthma symptoms and employment status (unemployed vs. full-time work and work disability vs. full-time work) in adults with asthma. Even after adjusting for age, gender, smoking and professional status, having more frequent asthma symptoms during last month or nightly wake-ups because of asthma was associated to undesirable employment status such as unemployment or work disability. Occurrence of persistent asthma symptoms was associated with unemployment and use of peroral corticosteroids was associated with work disability.

**Table 3:** Associations between different asthma symptoms^a^ and employment status. Crude logistic regression models and models adjusted for age, gender, smoking and professional status

		Unemployed vs. full-time work		Work disability vs. full-time work	
		Crude OR (95% CI)	Adjusted OR (95% CI)	Crude OR (95% CI)	Adjusted OR (95% CI)
Occurrence of asthma symptoms during last year	Persistently vs. none	3.2 (1.5–7.0)	2.4 (1.1–5.3)	3.9 (2.3–6.8)	1.6 (0.8–3.3)
Frequency of asthma symptoms during last month	Daily or almost daily vs. none	3.0 (1.7–5.2)	2.3 (1.3–4.2)	9.7 (6.1–15.4)	4.4 (2.3–8.2)
Frequency of asthma symptoms during last year	Daily or almost daily vs. none	4.2 (0.9–18.5)	3.0 (0.7–14.1)	3.4 (1.3–8.8)	1.8 (0.5–6.0)
Nightly wake-ups because of asthma symptoms during last month	> = 3 times a month vs. none	3.0 (2.1–4.3)	2.5 (1.7–3.8)	6.3 (4.6–8.7)	3.2 (2.1–4.8)
Nightly wake-ups because of asthma symptoms during last year	> = 3 times a month vs. none	2.6 (1.7–3.9)	2.4 (1.5–3.7)	4.4 (3.1–6.3)	2.6 (1.6–4.3)
Use of asthma medication during last year	Continuosly daily or almost daily vs. none	1.9 (1.0–3.4)	1.5 (0.8–2.9)	2.5 (1.5–4.2)	1.0 (0.5–2.0)
Subjects having used peroral corticoids because of asthma during last year		1.2 (0.9–1.7)	1.0 (0.7–1.5)	2.5 (1.9–3.3)	1.5 (1.1–2.2)

^a^Logistic regression analyses included calculations of odds ratios for less severe symptoms vs. no symptoms (e.g. seasonally vs. none and occasionally vs. none but data is shown only on worst symptoms vs. none

## Discussion

We found that among individuals with asthma, full-time work is associated with younger age, less symptomatic asthma despite of less asthma medication, nonmanual work and less smoking. Having frequent asthma symptoms or nightly wake-ups because of asthma is associated with less desirable employment status such as unemployment and work disability, even after adjusting for age, gender, smoking and professional status. Full-time work was interpreted as an indicator of successful coping in working life while all-cause sickness absence, disability pension applied or granted, unemployment or early retirement were interpreted as less desirable employment status, drifting away from working life, in this study. All subjects had clinically diagnosed asthma and the study covered all working-age adults with asthma in the city of Tampere, Finland, being well representative on population level.

Younger age was associated with full-time work. It may be independent of asthma, as younger people may be overall healthier without comorbidities. With just one health condition such as asthma, it may be easier to find ways to cope well at work compared to having multiple health issues. On the other hand, the tasks may differ between younger and older asthmatics as working life has changed and educational level is higher among younger people. Asthma may also be of different phenotypes in these age groups and therefore respond differently to current therapies [17].

We found that those who were working full time experienced less asthma symptoms altogether and also during leisure time than other groups. Also, when adjusted for age, gender, smoking and professional status, our results showed that having frequent asthma symptoms or nightly wake-ups because of asthma is associated with unemployment and work disability. This indicates that coping in working life may be more strongly affected by the severity of asthma rather than specific factors that may aggravate asthma symptoms at work. This supports earlier findings of severity of asthma predicting work disability [6, 12, 13], although one follow-up study of 111 workers with asthma concluded that adaptation to functional limitations played a major role in changes in sick leave in workers with asthma whereas lung function characteristics or disease history hardly played a role [18]. Some occupations such as cooks, gardeners, hairdressers and agricultural laborers still may be identified as “breath-taking jobs”, needing special attention for preventive measures [19]. This however does not explain why adults with asthma overall have disruptions in their working life, including respiratory sickness absences and all-cause long-term work disability [7, 10, 16]. A study from the U.S. compared the employment status of subjects with work-related and non-work-related asthma and found that being unable to work was associated with more frequent asthma symptoms and a very poorly controlled asthma [15]. Our results suggest the same phenomena showing associations between frequent symptoms or nightly wake-ups and undesirable employment status. In the U.S. study, asthma was self-reported whereas in our study, all subjects were reliably diagnosed for asthma. It is possible that in general, working environments have improved as indoor air factors and their effects on health have been recognized [20] and thereby on population level inherent severity of asthma is a more important factor in coping in working life than occupational exposures. It is noticeable that although workers who were working full time had less symptoms of asthma and their professional status was nonmanual, they did not use more regular asthma medication. On the contrary, they used less medication, which indicates that less desirable employment status is not associated to insufficient medical treatment of asthma.

Our study shows that individuals with asthma doing nonmanual work cope better in working life than those doing manual work. This may partially be explained by a cleaner work environment. We have found earlier, in line with other studies, that exposure to impurities such as dusts, chemicals or poor indoor air quality increases aggravation of asthma symptoms at work as does physically strenuous work [11, 21].

Although not always the case in individual level, educational levels are generally lower among workers doing manual work than nonmanual. Earlier studies have shown that lower education may increase the number of sickness absence days as much as 2.5-fold [8] and it has also been shown among asthmatics that lower level education increases the risk of work disability [6, 12]. Adaptation to functional limitations may play a major role in work ability [18] and the possibilities to adapt, adjust and have influence on one’s working environment are probably better among nonmanual workers.

There were more nonmanual workers in the full-time working group and the retired group compared to the groups with work disability and the unemployed, of whom about 60% of persons were manual workers. In these groups, a severe asthma and possible other health issues may affect their resilience in working life negatively. Asthma is the primary cause of disability pension in Finland in only less than 0,4% of disability pensions granted [22] whereas morbidity in musculoskeletal conditions and mental problems are leading causes of work disability [23]. A weakness of our study is the lack of information on co-morbidities and their possible effect on work ability.

The unemployed group was closest to full-time workers according to age and like full-time workers, they were younger than those with work disability and the retired. However, the unemployed group experienced more asthma symptoms, current smoking was more common and professional status was more likely to be manual than nonmanual worker than among full-time workers. In general, manual workers represent lower level education than nonmanual workers. Earlier studies have shown that unemployment among asthmatics is associated with not only lower education but also more severe asthma [5, 6]. It may be that the motivation to hold on to a manual job and perform well at work is decreased due to having more asthma symptoms.

Smoking aggravates asthma symptoms and decreases the effect of medication [24]. An 8-year follow-up study showed that improvements in working conditions and reducing smoking especially in the lower educational groups are likely to reduce the educational differences in sickness absence [8]. Eisner et al. showed that past smoking was associated with complete work disability but among the currently employed, smoking status was not associated with partial work disability [6]. In this study, the proportion of never-smokers and occasional smokers was greatest in the full-time working group and there were more ex-smokers in the work disability group than among full-time workers. It may be that with increasing asthma symptoms and decreasing quality of life due to symptoms, the motivation to quit smoking arises.

It could be assumed that physical exercise levels would be lower among those having most asthma symptoms compared to those having less symptoms. In our study however, there was no significant difference between the groups in physical exercise frequency. It may be that those who are active in working life have less time for physical exercise.

The strength of our study is that it is very well representative on population level. We recruited all working-age adults with asthma in Tampere city with established asthma and special reimbursement and diagnosis based on lung function criteria. Also, the response rate was high (79%). However, as a questionnaire-based study this lacks information on clinical measures and details of treatments on individual level. As a limitation of the study, we had data on all-cause sickness absence and lacked asthma-specific data. Also, there are limitations in the cross-sectional study design. As in all questionnaire-based studies, selection bias is possible and we do not know whether those who answered this questionnaire have e.g. more severe asthma or vice versa. The healthy worker effect, meaning e.g. that sicker individuals may be excluded from being hired or once hired, they may may seek transfer to less exposed jobs or leave work [25], may have contributed to selection bias in our study.

Our material was collected in year 2000 and some aspects in asthma treatment or working life may have changed. In Finland, our national asthma guidelines and asthma program have stressed active treatment with inhaled corticosteroids (ICS) since early 1990’s [26] and the use of ICS has not changed significantly since the collection of our data. Long-acting beta2-agonists (LABA) were introduced in mid- 1990’s but the use of this class of drugs has probably increased after year 2000 since the introduction of fixed ICS-LABA combinations. Finland has for long had a high level of social security and insurance system that has not significantly changed during the last decades and therefore our results from 2000 are still valid. The economic structure in Finland is typical for countries in western Europe and has not changed fundamentally after the year 2000.

## Conclusions

Among individuals with asthma, full-time workers are on average younger, more frequently nonmanual workers, they smoke less and have less asthma symptoms both at work and at leisure time despite of using less asthma medication. Even after adjusting for age, gender, smoking and professional status, logistic regression analyses show that having frequent asthma symptoms or nightly wake-ups because of asthma is associated with less desirable employment status such as unemployment and work disability. This suggests that in addition to following conventional procedures such as guideline-based asthma medication, smoking cessation and reduction of exposure to asthma triggers, it may be of great importance to support possibilities to change from manual to nonmanual work in preventing work disability and early exit from work.

## Additional file

Additional file 1: Appendix 1. Questions on employment status (work situation Q28), professional status (Q6) and asthma symptoms used in this study. (DOCX 15 kb)
