# Supplementary material for: Correlates of employment status in individuals with asthma: a cross-sectional survey
Source: J Occup Med Toxicol. 2017 Jul 24;12:19. doi: 10.1186/s12995-017-0165-6 (PMC5525303; doi:10.1186/s12995-017-0165-6)
Supplement: Additional file 1: — Appendix 1. Questions on employment status (work situation Q28), professional status (Q6) and asthma symptoms used in this study. (DOCX 15 kb) [file 12995_2017_165_MOESM1_ESM.docx]

Additional file 1. Questions on employment status (work situation Q28), professional status (Q6) and asthma symptoms used in this study.

28. What is your current work situation? You can circle several alternatives.

1. full-time work
2. part-time work
3. student
4. housework
5. maternity/paternity leave
6. retirement pending
7. sick leave
8. disability pension
9. other retirement
10. unemployed
11. laid off
12. other

6. Which of the following alternatives best describes the job you do or did last?

1. have not ever worked
2. am an entrepreneur
3. farmer/farmer’s wife
4. upper white-collar worker (administration, management, design, research and teaching tasks, etc.)
5. lower white-collar worker (foreman, clerical work, etc.)
6. blue-collar worker (industry, distribution and service workers and others in production)
7. housework, student
8. other

Questions on asthma symptoms

29. Have you had asthma symptoms during the last year (12 months)?

1. not at all
2. occasionally
3. seasonally
4. continually throughout the year, or at any time of year

31. How often on the average do you have asthma symptoms? Please answer both a and b.

not at all less than 1-2 times 3-5 times daily or

once a week a week a week almost daily

a) during the last month? 1 2 3 4 5

b) during the last year

(or if the symptoms

are seasonal, when the

symptom phase is underway) 1 2 3 4 5

32. How often, on the average, do you wake up at night with asthma symptoms? Please answer both a and b.

not at all less than 1-2 times 3-5 times daily or

once a week a week a week almost daily

a) during the last month? 1 2 3 4 5

b) during the last year

(or if the symptoms

are seasonal, when the

symptom phase is underway) 1 2 3 4 5

33. Have you used medication for your asthma during the last year (12 months)?

1. not at all
2. irregularly
3. seasonally (e.g. in the spring)
4. daily or almost daily

34. Have you used cortisone tablets to treat your asthma during the last year (12 months)?

1. no
2. yes
